# Mol­ecular structure, DFT studies and Hirshfeld analysis of anthracenyl chalcone derivatives

**DOI:** 10.1107/S2056989018006527

**Published:** 2018-05-04

**Authors:** Dian Alwani Zainuri, Ibrahim Abdul Razak, Suhana Arshad

**Affiliations:** a X-ray Crystallography Unit, School of Physics, Universiti Sains Malaysia, 11800 USM, Penang, Malaysia

**Keywords:** chalcone, crystal Structure, DFT, mol­ecular electrostatic potential, Hirshfeld surface

## Abstract

The mol­ecular and crystal structure of two new chalcone derivatives, (*E*)-1-(anthracen-9-yl)-3-[4-(piperidin-1-yl)phen­yl]prop-2-en-1-one, C_28_H_25_NO, (I), and (*E*)-1-(anthracen-9-yl)-3-[4-(di­phenyl­amino)­phen­yl]prop-2-en-1-one, C_35_H_25_NO, (II), with the fused-ring system at the same position are described. In the crystals of (I) and (II), the mol­ecules are linked *via* C—H⋯O hydrogen bonds into inversion dimers, forming *R*_2_^2^(22) and *R*_2_^2^(14) ring motifs, respectively. Weak inter­molecular C—H⋯π inter­actions further help to stabilize the crystal structure, forming a two-dimensional architecture. The mol­ecular structures are optimized using density functional theory (DFT) at B3LYP/6–311 G++(d,p) level and compared with the experimental results. The smallest HOMO–LUMO energy gaps of (I) (exp . 2.76 eV and DFT 3.40 eV) and (II) (exp . 2.70 eV and DFT 3.28 eV) indicates the suitability of these crystals in optoelectronic applications. All inter­molecular contacts and weaker contributions involved in the supra­molecular stabilization are investigated using Hirshfeld surface analysis. The mol­ecular electrostatic potential (MEP) further identifies the positive, negative and neutral electrostatic potential regions of the mol­ecules.

## Abstract 2

The structures of two new chalcone derivatives have been determined and are investigated using Hirshfeld surface analysis and mol­ecular electrostatic potential techniques.

## Chemical context   

Chalcone derivatives have attracted significant attention in the past few decades mainly because of their availability of high optical non-linearities resulting from the significant delocalization of the electron clouds throughout the chalcone system (D’silva *et al.*, 2011 ▸). A chalcone mol­ecule with a π-conjugated system provides a large charge-transfer axis with appropriate substituent groups on the two aromatic terminal rings. Furthermore, π-conjugated mol­ecular materials with fused rings are the focus of considerable inter­est in the emerging area of organic electronics, since the combination of excellent charge-carrier mobility and a high stability structure leads to potential optoelectronic applications (Wu *et al.*, 2010 ▸). As part of our studies in this area, the chalcone compounds (*E*)-1-(anthracen-9-yl)-3-[4-(piperidin-1-yl)phen­yl]prop-2-en-1-one, (I), and (*E*)-1-(anthracen-9-yl)-3-[4-(di­phenyl­amino)­phen­yl]prop-2-en-1-one, (II), were successfully synthesized and their crystal structures are reported herein.

## Structural commentary   

**Figure 1:**
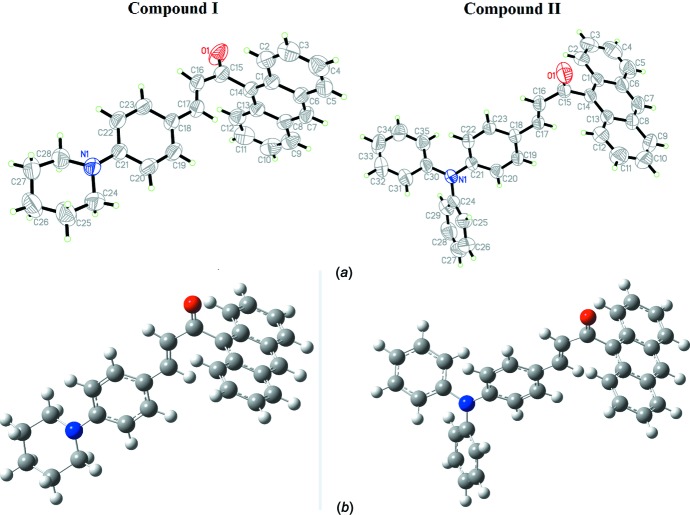
(a) The molecular structure of compounds (I) and (II) with 50% probability displacement ellipsoids. (b) The optimized structures of compounds (I) and (II) at the DFT/B3LYP 6–311++G(d,p) level.

The title compounds (I) and (II) (Fig. 1 ▸) crystallize in he triclinic and monoclinic space groups *P* 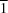 and *C2/c*, respectively. The bond lengths and angles are in normal ranges. The calculated values of compounds (I) and (II) determined from B3LYP/6-311G(d,p) calculations (given in the Supporting information) may provide information about the geometry of the mol­ecules. From the results, it can be concluded that this basis set is comparable in its approach to the experimental data. The slight deviations from the experimental values are due to the fact that the optimization is performed in an isolated condition, whereas the crystal environment and hydrogen-bonding inter­actions affect the results of the X-ray structure (Zainuri *et al.*, 2017 ▸).
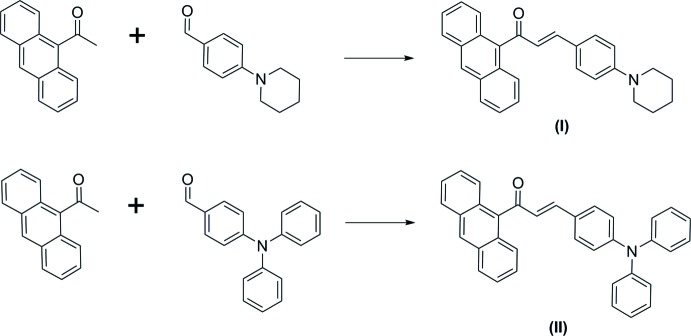


Compounds (I) and (II) contain an anthracene fused ring system and a 1-phenyl­piperidine or tri­phenyl­amine substituent, representing a *D*–π–*D* inter­molecular charge-transfer system. The piperidine ring (N1/C24–C28) in (I) adopts a chair conformation with puckering parameters *Q* = 0.521 (4), Θ = 3.1 (3)° and φ = 221 (6)°. The enone moiety (O1/C15–C17) in compounds (I) and (II) adopts an *s-trans* configuration with respect to the C15=O1 and C16=C17 bonds. Both compounds (I) and (II) are twisted at the C14—C15 bonds with C1—C14—C15—C16 torsion angles of 101.5 (3) and 93.66 (18)°, respectively. The corresponding torsion angles from the DFT study are 88.68 and 90.29°. In addition, the C17—C18 bond is also twisted slightly in (I) and (II) with the C16—C17—C18—C19 torsion angles being 171.5 (3)° (Exp) and 179.22° (DFT) in (I) and −164.77 (16)° (Exp) and 175.94° (DFT) in (II). The torsion angle difference between the experimental and DFT studies are due to the formation of inter­molecular inter­actions involving the anthracene fused-ring system and the terminal substituent of the 1-phenyl­piperidine and tri­phenyl­amine units. The observed inter­molecular inter­actions in the crystal packing are the main cause of the angle deviation between the experimental and the theoretical results.

The enone moiety for (I) [O1/C15–C17, maximum deviation of 0.052 (3) Å at C16] forms dihedral angles of 82.9 (3), 12.0 (3) and 8.1 (3)° with the anthracene ring system (C1–C14), the benzene ring (C18-C23) and the piperidine ring (N1/C24–C28), respectively. The anthracene ring system forms dihedral angles of 86.74 (10) and 85.55 (12)° with the 1-phenyl­piperidine rings C18–C23 and N1/C24–C28, respectively. Meanwhile, in compound (II), the enone moiety [O1/ C15–C17, maximum deviation of 0.0287 (15) Å at C16] forms dihedral angles of 87.30 (16), 17.13 (16), 72.55 (17) and 79.16 (16)° with the anthracene ring system (C1–C14) and the benzene rings C18–C23, C24–C29 and C30–C35, respectively. The dihedral angle between the anthracene ring system and the tri­phenyl­amine benzene rings C18–C23, C24–C29 and C30–C35 are 75.86 (6), 79.81 (8) and 12.84 (8)°, respectively. The large dihedral-angle deviation indicates that the possibility for electronic effects between the anthracene units through the enone moiety has decreased (Jung *et al.*, 2008 ▸). Furthermore, the bulkiness of the anthracene ring system gives rise to a highly twisted structure for both compounds (Zainuri *et al.*, 2018*a* ▸,*b* ▸).

## Supra­molecular features   

**Figure 2:**
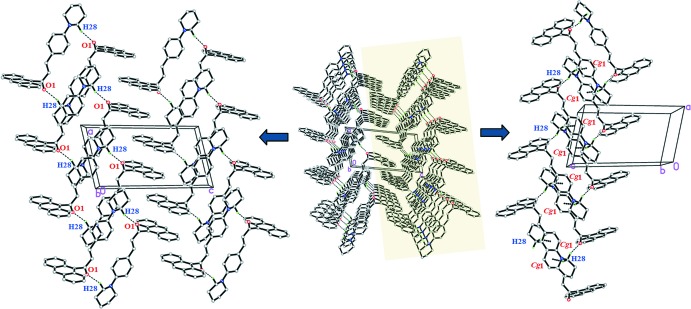
The crystal packing of (I) showing weak C—H⋯O and C—H⋯π inter­actions.

In the crystal packing of compound (I), the mol­ecules are connected *via* inter­molecular C28—H28*B*⋯O1^i^ inter­actions (Table 1 ▸), forming inversion dimers with 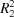
(22) ring motifs. These ring motifs further link into one-dimensional columns along the *b*-axis direction (Fig. 2 ▸). The crystal packing is stabilized by weak C28—H28*A*⋯ *Cg*1^ii^ inter­actions (Table 1 ▸). Together, these inter­actions connect the mol­ecules into sheets parallel to the *ac* plane.

**Figure 3:**
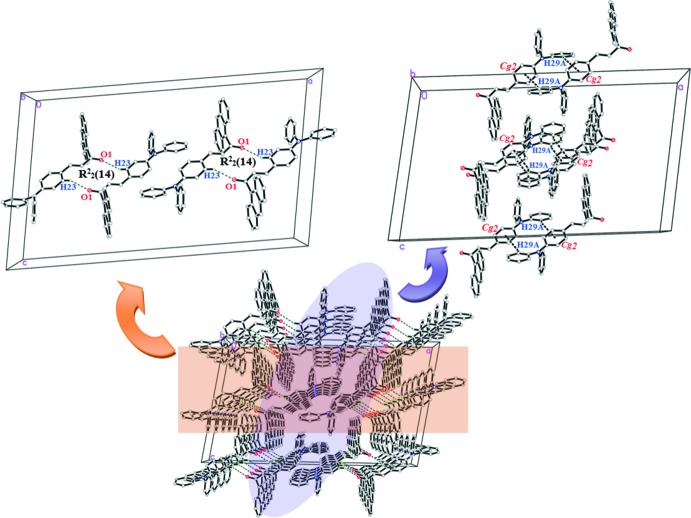
The weak C—H⋯ O and C—H⋯π inter­actions in compound (II).

Similary, in compound (II), C23—H23*A*⋯ O1^i^ (Table 1 ▸ and Fig. 3 ▸) hydrogen bonds connect the mol­ecules into centrosymmetric dimers, forming 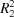
(14) ring motifs. These dimers are further linked into infinite columns along the *c*-axis direction. C29—H29*A*⋯ *Cg*1^ii^ inter­actions (Table 2 ▸) are also observed. As in (I), the crystal structure comprises sheets parallel to the *ac* plane.

## UV–Vis absorption analysis   

**Figure 4:**
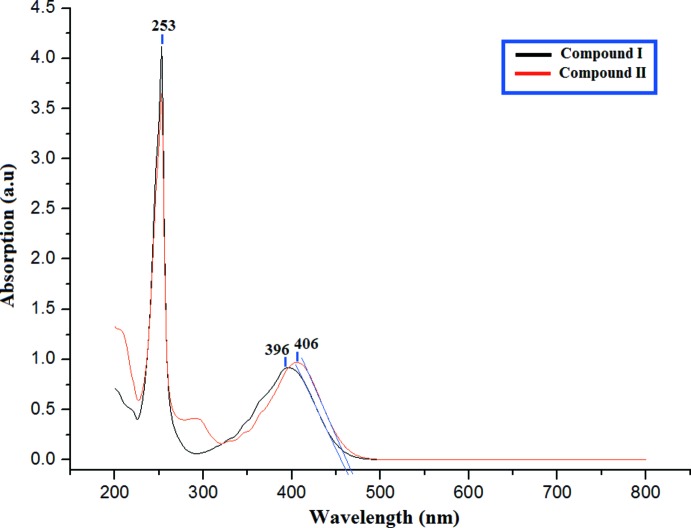
UV–Vis absorption spectra for compounds (I) and (II).

The strongest absorption and smaller energy gap, particularly in the visible region, is important feature in the suitability for optoelectronic application. The electronic absorption and excitation properties of (I) and (II) were estimated theoretically by applying the time-dependent DFT approach at the B3LYP level of theory with the 6-311++G(d,p) basis set. The experimental absorptions (Fig. 4 ▸) of (I) and (II) are reported at 396 and 406 nm, while simulated values are observed at 397 and 415 nm, respectively. The theoretical wavelengths are shifted to higher wavelengths because the calculations are confined to the gaseous equivalent whereas the observations are from the solution state.

**Figure 5:**
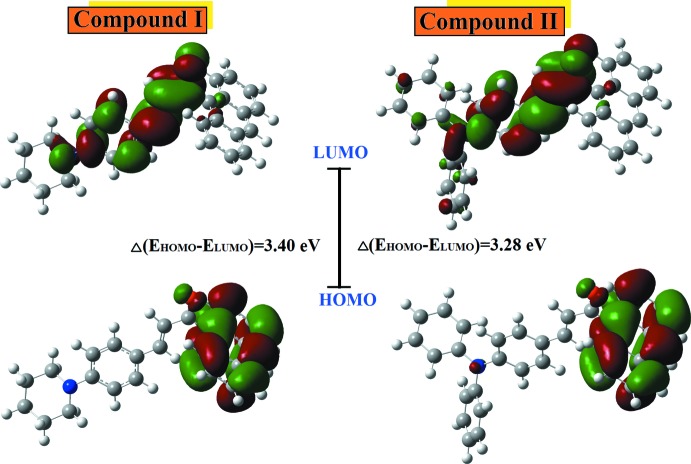
The electron distribution of the HOMO and LUMO energy levels of (I) and (II).

The experimental energy band gaps for (I) and (II) are 2.76 and 2.70 eV, respectively, through an extrapolation of the linear trend. The calculations of the mol­ecular orbital geometry show that the absorption maxima of the mol­ecules correspond to the electron transition between the frontier orbitals highest occupied mol­ecular orbital (HOMO) to the lowest unoccupied mol­ecular orbital (LUMO) (Fig. 5 ▸). The predicted energy gaps for compounds (I) and (II) are 3.40 and 3.28 eV, respectively. The small HOMO–LUMO energy gap in these compounds shows the chemical reactivity is stronger and the kinetic stability is weaker, which in turn increase the polarizability and NLO activity (Maidur *et al.*, 2018 ▸).

## Hirshfeld surface analysis   

**Figure 6:**
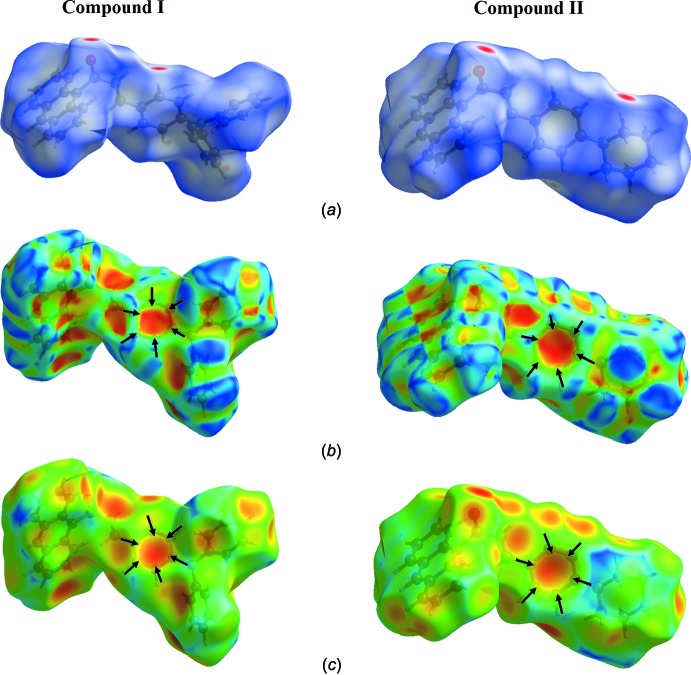
View of the Hirshfeld Surfaces, showing (*a*) *d*_norm_ with the red spot showing the involvement of the C—H⋯O inter­actions, (*b*) mapped over shape-index and (*c*) mapped over *d*_e_ with the pale-orange spot inside the black arrows indicating the C—H⋯π inter­actions.

Hirshfeld surface analysis assigns inter­molecular inter­actions inside the unit-cell packing. The *d_norm_*, shape-index and *d_e_* (Wolff *et al.*, 2012 ▸) surfaces are presented in Fig. 6 ▸ *a*, *b* and *c*, respectively. All C—H⋯ O and C—H⋯π contacts are recognized in the *d_norm_* mapped surface as deep-red depression areas in Fig. 6 ▸ *a*. The C—H⋯ O contacts are observed in both compounds (I) and (II). The presence of C—H⋯π inter­actions is indicated through the combination of pale-orange and bright-red spots, which are present on the shape-index surface, identified with black arrows (Fig. 6 ▸ *b*).

**Figure 7:**
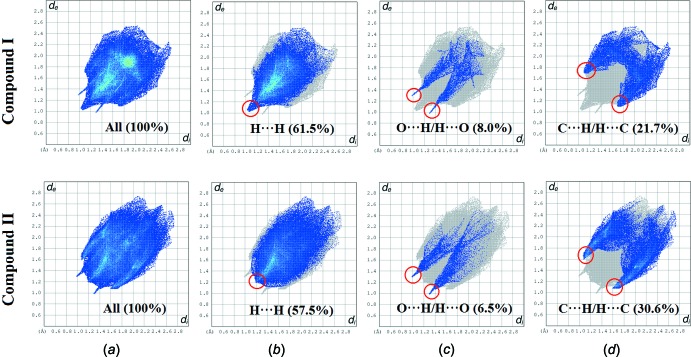
Fingerprint plots of inter­actions, listing the percentage of contacts (*a*) full two-dimensional fingerprint plots; (*b*) H⋯H (*c*) O⋯H/H⋯O and (*d*) C⋯H/H⋯C contributions to the total Hirshfeld surface. The outline of the full fingerprint plots is shown in grey.

Two-dimensional fingerprint plots as shown in Fig. 7 ▸. These illustrate the difference between the inter­molecular inter­action patterns and the major inter­molecular contacts associated in both compounds. The H⋯H contacts appear to be the major contributor to the Hirshfeld surface; these are shown in Fig. 7 ▸ *b* as one distinct spike with a minimum value *d_e_* + *d_i_* that is approximately less than the sum of van der Waals radii (2.4 Å). Furthermore, the inter­molecular C—H⋯π inter­actions for compounds (I) and (II) are characterized by the short inter­atomic C⋯H/H⋯C contacts with percentage contributions of 21.7% (I) and 30.6% (II), showing two distinct spikes with *d_e_* + *d_i_* ∼2.8 Å (I) and 2.7 Å (II). Additionally, the O⋯H/H⋯O contacts indicate the presence of inter­molecular C—H⋯ O inter­actions with percentage contributions of 8.0% (I) and 6.5% (II) and are indicated by a pair of wings at *d_e_* + *d_i_* ∼2.3 Å (Fig. 7 ▸ *c*).

## Mol­ecular Electrostatic Potential   

**Figure 8:**
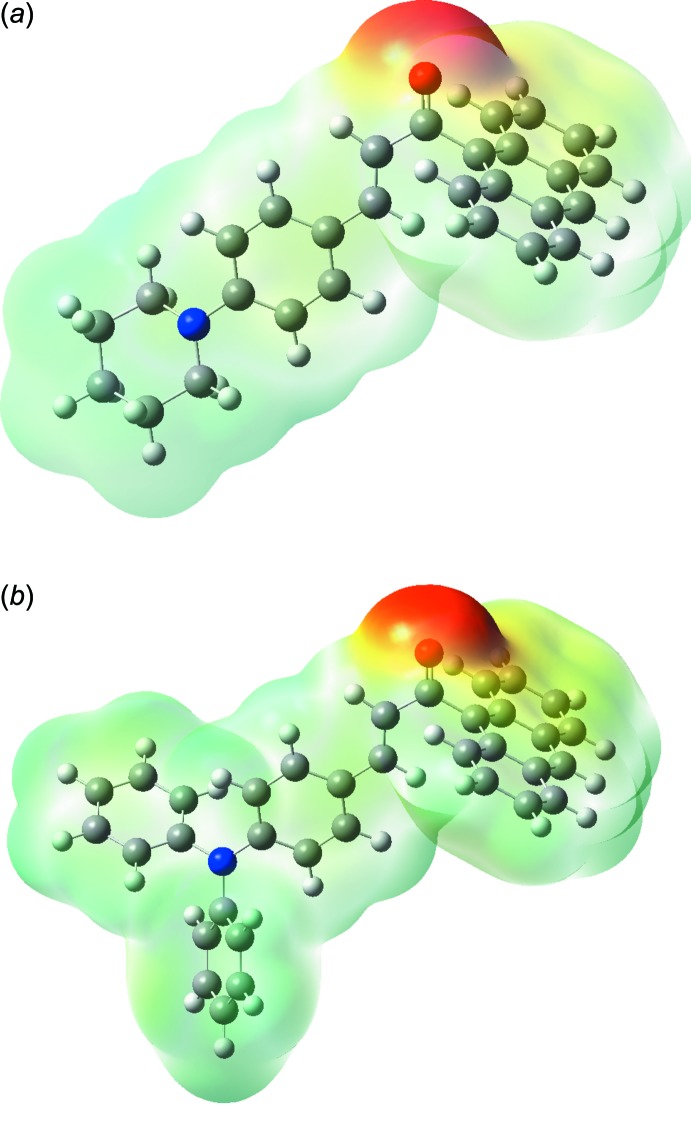
The total electron density three-dimensional surface mapped for (*a*) compound (I) and (*b*) compound (II) with the electrostatic potential calculated at the B3LYP/6–311 G++ (d,p) level.

The mol­ecular electrostatic potential (MEP) has become firmly established as an effective guide to mol­ecular inter­actions. The importance of MEPs lies in the fact that it simultaneously displays mol­ecular size and shape, as well as positive, negative and neutral electrostatic potential regions, in terms of colour grading and is useful in suties of the mol­ecular structure and its physicochemical property relationship (Murray & Sen, 1996 ▸; Scrocco & Tomasi, 1978 ▸). The MEP maps of (I) and (II) mol­ecules were calculated theoretically at the B3LYP/6-311G++(d,p) level of theory and the obtained plots are shown in Fig. 8 ▸. The red-coloured region is nucleophile and electron rich, whereas the blue colour indicates the electrophile region with poor electrons in the vicinity, and the remaining white region shows the neutrality of atoms. These sites given information about the region from where the mol­ecule can have inter­molecular inter­actions (Gunasekaran & Srinivasan, 2008 ▸).

In (I) and (II), the reactive sites are near the C=O group; this is the region having the most negative potential spots (red colour), all over the oxygen atom due to the C—H⋯ O inter­actions in the crystal structure. The negative potential values of compounds (I) and (II) of −0.06268 and −0.06453 a.u. indicate the strongest repulsion (electrophilic attack). Meanwhile, the most positive regions for (I) and (II) are localized on the hydrogen atoms and show the strongest attraction (nucleophilic attack) sites involving the anthrancene group and its subtsituent groups of the 1-phenyl­piperidine (I) and tri­phenyl­amine (II) moieties.

## Database survey   

A survey of Cambridge Structural Database (CSD, Version 5.38, last update Nov 2016; Groom *et al.*, 2016 ▸) revealed fused-ring substituted chalcones similar to (I) and (II). There are four compounds that have ananthracene–ketone substituent on the chalcone: 9-anthryl styryl ketone and 9,10-anthryl bis­(styryl ketone) (Harlow *et al.*, 1975 ▸), (*2E*)-1-(anthracen-9-yl)-3-[4-(propan-2-yl)phen­yl]prop-2-en-1-one (Girisha *et al.*, 2016 ▸) and (*E*)-1-(anthracen-9-yl)-3-(2-chloro-6-fluoro­phen­yl)prop-2-en- 1-one (Abdullah *et al.*, 2016 ▸). Zainuri *et al.*, 20182018*a* ▸,*b* ▸) reported two anthracene substituents on the chalcone (*E*)-1,3-bis­(anthracen-9-yl)prop-2-en-1-one. Other related compounds include 1-(anthracen-9-yl)-2-methyl­prop-2-en-1-one (Agrahari *et al.*, 2015 ▸) and 9-anthroylacetone (Cicogna *et al.*, 2004 ▸).

## Synthesis and crystallization   

A mixture of 9-acetyl­anthrancene (0.5 mmol) and 4-(piperidin-1-yl)benzaldehyde (0.5 mmol) and 4-(di­phenyl­amino)­benzaldehyde (0.5 mmol) for compound (I) and (II), respectively, was dissolved in methanol (20 ml). A catalytic amount of NaOH (5 ml, 20%) was added to the solution dropwise with vigorous stirring. The reaction mixture was stirred for about 5–6 h at room temperature. After stirring, the contents of the flask were poured into ice-cold water (50 ml). The resultant crude products were filtered, washed successively with distilled water and recrystallized to get the corresponding chalcones. Crystals suitable for X-ray diffraction were obtained by the slow evaporation technique from acetone.

## Refinement   

Crystal data collection and structure refinement details are summarized in Table 3 ▸. All H atoms were positioned geometrically [C—H = 0.93 and 0.97 Å (in (I)) and 0.93 Å (in (II))] and refined using riding model with *U*_iso_(H) = 1.2*U*_eq_(C).

## Supplementary Material

Crystal structure: contains datablock(s) I, II. DOI: 10.1107/S2056989018006527/lh5873sup1.cif

Structure factors: contains datablock(s) I. DOI: 10.1107/S2056989018006527/lh5873Isup2.hkl

Structure factors: contains datablock(s) II. DOI: 10.1107/S2056989018006527/lh5873IIsup3.hkl

Comparison between selected calculated (DFT) and experimental geometrical data. DOI: 10.1107/S2056989018006527/lh5873sup4.pdf

CCDC references: 1824550, 1817218

Additional supporting information:  crystallographic information; 3D view; checkCIF report

## Figures and Tables

**Table 1:** Hydrogen-bond geometry (Å, °) for (I) *Cg*1 is the centroid of the C18–C23 ring.

*D*—H⋯*A*	*D*—H	H⋯*A*	*D*⋯*A*	*D*—H⋯*A*
C28—H28*B*⋯O1^i^	0.97	2.36	3.262 (4)	154
C28—H28*A*⋯*Cg*1^ii^	0.97	2.95	3.861 (4)	157

Symmetry codes: (i) 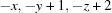; (ii) 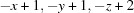.

**Table 2:** Hydrogen-bond geometry (Å, °) for (II) *Cg*1 is the centroid of the C18–C23 ring.

*D*—H⋯*A*	*D*—H	H⋯*A*	*D*⋯*A*	*D*—H⋯*A*
C23—H23*A*⋯O1^i^	0.93	2.40	3.221 (2)	147
C29—H29*A*⋯*Cg*1^ii^	0.93	2.96	3.739 (19)	142

Symmetry codes: (i) 
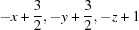
; (ii) 
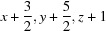
.

**Table 3:** Experimental details

	(I)	(II)
Crystal data		
Chemical formula	C_28_H_25_NO	C_35_H_25_NO
*M*_r_	391.49	475.56
Crystal system, space group	Triclinic, *P* 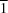	Monoclinic, *C*2/*c*
Temperature (K)	296	296
*a*, *b*, *c* (Å)	8.0535 (15), 9.0457 (17), 15.352 (3)	31.2875 (16), 9.0470 (4), 18.3643 (8)
α, β, γ (°)	106.553 (4), 101.572 (4), 94.385 (4)	90, 99.388 (3), 90
*V* (Å^3^)	1039.6 (3)	5128.5 (4)
*Z*	2	8
Radiation type	Mo *K*α	Mo *K*α
μ (mm^−1^)	0.08	0.07
Crystal size (mm)	0.64 × 0.23 × 0.10	0.96 × 0.23 × 0.17

Data collection		
Diffractometer	Bruker SMART APEXII DUO CCD area-detector	Bruker SMART APEXII DUO CCD area-detector
Absorption correction	Multi-scan (*SADABS*; Bruker, 2009 ▸)	Multi-scan (*SADABS*; Bruker, 2009 ▸)
*T*_min_, *T*_max_	0.724, 0.972	0.645, 0.957
No. of measured, independent and observed [*I* > 2σ(*I*)] reflections	27976, 4812, 2122	98729, 7726, 4183
*R*_int_	0.079	0.076
(sin θ/λ)_max_ (Å^−1^)	0.652	0.712

Refinement		
*R*[*F* ^2^ > 2σ(*F* ^2^)], *wR*(*F* ^2^), *S*	0.068, 0.246, 1.01	0.057, 0.144, 1.02
No. of reflections	4812	7726
No. of parameters	271	334
H-atom treatment	H-atom parameters constrained	H-atom parameters constrained
Δρ_max_, Δρ_min_ (e Å^−3^)	0.26, −0.19	0.13, −0.14

Computer programs: *APEX2* and *SAINT* (Bruker, 2009 ▸), *SHELXL2014* and *SHELXL2013* (Sheldrick, 2015 ▸), *SHELXTL* (Sheldrick, 2008 ▸) and *PLATON* (Spek, 2009 ▸).

## supplementary crystallographic information

### (E)-1-(Anthracen-9-yl)-3-[4-(piperidin-1-yl)phenyl]prop-2-en-1-one (I) Crystal data

**Table:**

C_28_H_25_NO	*Z* = 2
*M**_r_* = 391.49	*F*(000) = 416
Triclinic, *P*1	*D*_x_ = 1.251 Mg m^−^^3^
*a* = 8.0535 (15) Å	Mo *K*α radiation, λ = 0.71073 Å
*b* = 9.0457 (17) Å	Cell parameters from 1764 reflections
*c* = 15.352 (3) Å	θ = 2.4–19.6°
α = 106.553 (4)°	µ = 0.08 mm^−^^1^
β = 101.572 (4)°	*T* = 296 K
γ = 94.385 (4)°	Plate, yellow
*V* = 1039.6 (3) Å^3^	0.64 × 0.23 × 0.10 mm

### (E)-1-(Anthracen-9-yl)-3-[4-(piperidin-1-yl)phenyl]prop-2-en-1-one (I) Data collection

**Table:**

Bruker SMART APEXII DUO CCD area-detector diffractometer	2122 reflections with *I* > 2σ(*I*)
Radiation source: fine-focus sealed tube	*R*_int_ = 0.079
φ and ω scans	θ_max_ = 27.6°, θ_min_ = 1.4°
Absorption correction: multi-scan (*SADABS*; Bruker, 2009)	*h* = −10→10
*T*_min_ = 0.724, *T*_max_ = 0.972	*k* = −11→11
27976 measured reflections	*l* = −19→19
4812 independent reflections	

### (E)-1-(Anthracen-9-yl)-3-[4-(piperidin-1-yl)phenyl]prop-2-en-1-one (I) Refinement

**Table:**

Refinement on *F*^2^	0 restraints
Least-squares matrix: full	Hydrogen site location: inferred from neighbouring sites
*R*[*F*^2^ > 2σ(*F*^2^)] = 0.068	H-atom parameters constrained
*wR*(*F*^2^) = 0.246	*w* = 1/[σ^2^(*F*_o_^2^) + (0.115*P*)^2^ + 0.0669*P*] where *P* = (*F*_o_^2^ + 2*F*_c_^2^)/3
*S* = 1.01	(Δ/σ)_max_ < 0.001
4812 reflections	Δρ_max_ = 0.26 e Å^−^^3^
271 parameters	Δρ_min_ = −0.19 e Å^−^^3^

### (E)-1-(Anthracen-9-yl)-3-[4-(piperidin-1-yl)phenyl]prop-2-en-1-one (I) Special details

**Table:**

Experimental. The following wavelength and cell were deduced by SADABS from the direction cosines etc. They are given here for emergency use only: CELL 0.71134 9.070 15.379 16.135 101.576 94.356 106.571
Geometry. All esds (except the esd in the dihedral angle between two l.s. planes) are estimated using the full covariance matrix. The cell esds are taken into account individually in the estimation of esds in distances, angles and torsion angles; correlations between esds in cell parameters are only used when they are defined by crystal symmetry. An approximate (isotropic) treatment of cell esds is used for estimating esds involving l.s. planes.

### (E)-1-(Anthracen-9-yl)-3-[4-(piperidin-1-yl)phenyl]prop-2-en-1-one (I) Fractional atomic coordinates and isotropic or equivalent isotropic displacement parameters (Å^2^)

**Table:**

	*x*	*y*	*z*	*U*_iso_*/*U*_eq_	
O1	−0.3955 (3)	0.4115 (3)	0.73584 (14)	0.0949 (8)	
N1	0.6347 (3)	0.8072 (2)	1.09764 (14)	0.0575 (6)	
C1	−0.2379 (3)	0.4582 (3)	0.57411 (17)	0.0542 (6)	
C2	−0.1842 (3)	0.3116 (3)	0.5702 (2)	0.0694 (8)	
H1	−0.1747	0.2771	0.6224	0.083*	
C3	−0.1466 (4)	0.2206 (4)	0.4920 (2)	0.0848 (9)	
H2	−0.1098	0.1253	0.4912	0.102*	
C4	−0.1627 (4)	0.2695 (4)	0.4119 (2)	0.0884 (10)	
H3	−0.1375	0.2059	0.3583	0.106*	
C5	−0.2139 (4)	0.4060 (4)	0.4123 (2)	0.0788 (9)	
H4	−0.2249	0.4360	0.3585	0.095*	
C6	−0.2517 (3)	0.5066 (3)	0.49269 (16)	0.0575 (7)	
C7	−0.3004 (3)	0.6497 (3)	0.49477 (18)	0.0632 (7)	
H5	−0.3109	0.6799	0.4411	0.076*	
C8	−0.3343 (3)	0.7503 (3)	0.57345 (18)	0.0579 (7)	
C9	−0.3802 (3)	0.8996 (4)	0.5773 (2)	0.0726 (8)	
H6	−0.3851	0.9339	0.5253	0.087*	
C10	−0.4167 (4)	0.9924 (4)	0.6537 (3)	0.0812 (9)	
H10	−0.4481	1.0895	0.6542	0.097*	
C11	−0.4080 (4)	0.9442 (4)	0.7330 (2)	0.0791 (9)	
H11	−0.4349	1.0089	0.7857	0.095*	
C12	−0.3612 (3)	0.8049 (3)	0.73383 (19)	0.0657 (7)	
H12	−0.3546	0.7758	0.7877	0.079*	
C13	−0.3216 (3)	0.7014 (3)	0.65471 (16)	0.0533 (6)	
C14	−0.2753 (3)	0.5560 (3)	0.65304 (16)	0.0529 (6)	
C15	−0.2738 (3)	0.5015 (3)	0.73695 (17)	0.0604 (7)	
C16	−0.1294 (3)	0.5534 (3)	0.81633 (17)	0.0610 (7)	
H16	−0.1401	0.5308	0.8705	0.073*	
C17	0.0172 (3)	0.6311 (3)	0.81710 (17)	0.0550 (6)	
H17	0.0211	0.6584	0.7635	0.066*	
C18	0.1718 (3)	0.6790 (3)	0.89032 (16)	0.0519 (6)	
C19	0.3209 (3)	0.7416 (3)	0.87506 (18)	0.0667 (8)	
H19	0.3190	0.7560	0.8173	0.080*	
C20	0.4712 (3)	0.7833 (3)	0.94162 (18)	0.0680 (8)	
H20	0.5679	0.8259	0.9279	0.082*	
C21	0.4836 (3)	0.7640 (3)	1.02901 (16)	0.0516 (6)	
C22	0.3322 (3)	0.7030 (3)	1.04536 (17)	0.0610 (7)	
H22	0.3334	0.6892	1.1032	0.073*	
C23	0.1831 (3)	0.6632 (3)	0.97861 (17)	0.0611 (7)	
H23	0.0850	0.6240	0.9927	0.073*	
C24	0.7882 (3)	0.8619 (4)	1.0742 (2)	0.0757 (9)	
H24A	0.7613	0.9339	1.0389	0.091*	
H24B	0.8275	0.7740	1.0346	0.091*	
C25	0.9284 (4)	0.9408 (4)	1.1579 (2)	0.0930 (11)	
H25A	0.8976	1.0392	1.1913	0.112*	
H25B	1.0317	0.9634	1.1378	0.112*	
C26	0.9649 (4)	0.8453 (4)	1.2232 (2)	0.0958 (11)	
H26A	1.0141	0.7547	1.1941	0.115*	
H26B	1.0468	0.9066	1.2800	0.115*	
C27	0.8026 (4)	0.7947 (4)	1.2463 (2)	0.0897 (10)	
H27A	0.8248	0.7249	1.2836	0.108*	
H27B	0.7629	0.8850	1.2832	0.108*	
C28	0.6666 (4)	0.7140 (4)	1.1604 (2)	0.0755 (9)	
H28A	0.7004	0.6167	1.1277	0.091*	
H28B	0.5614	0.6895	1.1782	0.091*	

### (E)-1-(Anthracen-9-yl)-3-[4-(piperidin-1-yl)phenyl]prop-2-en-1-one (I) Atomic displacement parameters (Å^2^)

**Table:**

	*U*^11^	*U*^22^	*U*^33^	*U*^12^	*U*^13^	*U*^23^
O1	0.0856 (14)	0.1238 (18)	0.0697 (13)	−0.0401 (13)	0.0025 (11)	0.0461 (13)
N1	0.0538 (12)	0.0678 (14)	0.0501 (12)	0.0009 (10)	0.0081 (10)	0.0215 (10)
C1	0.0478 (14)	0.0621 (17)	0.0490 (15)	−0.0043 (12)	0.0073 (11)	0.0170 (13)
C2	0.0658 (18)	0.0722 (19)	0.0683 (19)	−0.0012 (15)	0.0129 (15)	0.0236 (15)
C3	0.085 (2)	0.069 (2)	0.090 (2)	0.0049 (16)	0.0221 (19)	0.0092 (19)
C4	0.091 (2)	0.093 (3)	0.066 (2)	−0.003 (2)	0.0264 (18)	−0.0005 (19)
C5	0.080 (2)	0.094 (2)	0.0528 (17)	−0.0077 (19)	0.0148 (15)	0.0124 (17)
C6	0.0506 (14)	0.0739 (18)	0.0416 (14)	−0.0052 (13)	0.0062 (11)	0.0145 (13)
C7	0.0575 (16)	0.088 (2)	0.0448 (15)	−0.0011 (15)	0.0026 (12)	0.0307 (15)
C8	0.0485 (14)	0.0717 (18)	0.0531 (16)	−0.0001 (13)	0.0063 (12)	0.0249 (14)
C9	0.0624 (17)	0.084 (2)	0.078 (2)	0.0066 (16)	0.0074 (15)	0.0418 (18)
C10	0.0686 (19)	0.077 (2)	0.096 (3)	0.0075 (16)	0.0124 (18)	0.027 (2)
C11	0.0720 (19)	0.079 (2)	0.075 (2)	0.0070 (17)	0.0159 (16)	0.0079 (17)
C12	0.0608 (17)	0.0736 (19)	0.0567 (17)	−0.0009 (14)	0.0121 (13)	0.0146 (15)
C13	0.0425 (13)	0.0679 (17)	0.0446 (14)	−0.0048 (12)	0.0042 (11)	0.0169 (13)
C14	0.0476 (14)	0.0642 (16)	0.0433 (14)	−0.0082 (12)	0.0044 (11)	0.0196 (12)
C15	0.0612 (16)	0.0677 (17)	0.0519 (15)	−0.0027 (14)	0.0111 (13)	0.0223 (13)
C16	0.0687 (17)	0.0704 (17)	0.0447 (14)	−0.0022 (14)	0.0075 (13)	0.0257 (13)
C17	0.0633 (16)	0.0592 (15)	0.0445 (14)	0.0049 (13)	0.0091 (12)	0.0221 (12)
C18	0.0569 (15)	0.0563 (15)	0.0434 (14)	0.0056 (12)	0.0109 (12)	0.0178 (11)
C19	0.0674 (17)	0.088 (2)	0.0476 (15)	−0.0017 (15)	0.0129 (14)	0.0283 (14)
C20	0.0586 (16)	0.092 (2)	0.0555 (17)	−0.0045 (15)	0.0151 (14)	0.0292 (15)
C21	0.0545 (15)	0.0567 (15)	0.0450 (14)	0.0055 (12)	0.0129 (12)	0.0178 (11)
C22	0.0630 (17)	0.0770 (18)	0.0419 (14)	−0.0010 (14)	0.0112 (13)	0.0205 (13)
C23	0.0563 (15)	0.0764 (18)	0.0519 (15)	−0.0021 (13)	0.0145 (13)	0.0232 (13)
C24	0.0600 (17)	0.095 (2)	0.073 (2)	−0.0034 (16)	0.0141 (15)	0.0316 (17)
C25	0.0672 (19)	0.103 (2)	0.105 (3)	−0.0109 (18)	−0.0082 (19)	0.052 (2)
C26	0.065 (2)	0.109 (3)	0.108 (3)	−0.0009 (18)	−0.0116 (18)	0.049 (2)
C27	0.074 (2)	0.122 (3)	0.076 (2)	0.0079 (19)	−0.0034 (17)	0.051 (2)
C28	0.0664 (18)	0.087 (2)	0.078 (2)	0.0026 (15)	0.0066 (15)	0.0422 (17)

### (E)-1-(Anthracen-9-yl)-3-[4-(piperidin-1-yl)phenyl]prop-2-en-1-one (I) Geometric parameters (Å, º)

**Table:**

O1—C15	1.219 (3)	C15—C16	1.442 (3)
N1—C21	1.386 (3)	C16—C17	1.323 (3)
N1—C24	1.445 (3)	C16—H16	0.9300
N1—C28	1.449 (3)	C17—C18	1.442 (3)
C1—C14	1.385 (3)	C17—H17	0.9300
C1—C2	1.415 (4)	C18—C19	1.379 (3)
C1—C6	1.425 (3)	C18—C23	1.389 (3)
C2—C3	1.354 (4)	C19—C20	1.366 (3)
C2—H1	0.9300	C19—H19	0.9300
C3—C4	1.406 (4)	C20—C21	1.387 (3)
C3—H2	0.9300	C20—H20	0.9300
C4—C5	1.331 (4)	C21—C22	1.398 (3)
C4—H3	0.9300	C22—C23	1.361 (3)
C5—C6	1.413 (4)	C22—H22	0.9300
C5—H4	0.9300	C23—H23	0.9300
C6—C7	1.375 (4)	C24—C25	1.486 (4)
C7—C8	1.380 (4)	C24—H24A	0.9700
C7—H5	0.9300	C24—H24B	0.9700
C8—C9	1.415 (4)	C25—C26	1.500 (4)
C8—C13	1.426 (3)	C25—H25A	0.9700
C9—C10	1.336 (4)	C25—H25B	0.9700
C9—H6	0.9300	C26—C27	1.493 (4)
C10—C11	1.396 (4)	C26—H26A	0.9700
C10—H10	0.9300	C26—H26B	0.9700
C11—C12	1.345 (4)	C27—C28	1.491 (4)
C11—H11	0.9300	C27—H27A	0.9700
C12—C13	1.417 (3)	C27—H27B	0.9700
C12—H12	0.9300	C28—H28A	0.9700
C13—C14	1.389 (3)	C28—H28B	0.9700
C14—C15	1.503 (3)		
			
C21—N1—C24	118.4 (2)	C16—C17—C18	128.4 (2)
C21—N1—C28	117.4 (2)	C16—C17—H17	115.8
C24—N1—C28	113.0 (2)	C18—C17—H17	115.8
C14—C1—C2	123.1 (2)	C19—C18—C23	115.7 (2)
C14—C1—C6	119.1 (2)	C19—C18—C17	120.8 (2)
C2—C1—C6	117.7 (2)	C23—C18—C17	123.5 (2)
C3—C2—C1	121.2 (3)	C20—C19—C18	122.5 (2)
C3—C2—H1	119.4	C20—C19—H19	118.8
C1—C2—H1	119.4	C18—C19—H19	118.8
C2—C3—C4	120.3 (3)	C19—C20—C21	121.9 (2)
C2—C3—H2	119.9	C19—C20—H20	119.1
C4—C3—H2	119.9	C21—C20—H20	119.1
C5—C4—C3	120.5 (3)	N1—C21—C20	122.6 (2)
C5—C4—H3	119.7	N1—C21—C22	121.5 (2)
C3—C4—H3	119.7	C20—C21—C22	115.9 (2)
C4—C5—C6	121.4 (3)	C23—C22—C21	121.6 (2)
C4—C5—H4	119.3	C23—C22—H22	119.2
C6—C5—H4	119.3	C21—C22—H22	119.2
C7—C6—C5	122.0 (3)	C22—C23—C18	122.5 (2)
C7—C6—C1	119.2 (2)	C22—C23—H23	118.8
C5—C6—C1	118.8 (3)	C18—C23—H23	118.8
C6—C7—C8	122.5 (2)	N1—C24—C25	112.8 (2)
C6—C7—H5	118.7	N1—C24—H24A	109.0
C8—C7—H5	118.7	C25—C24—H24A	109.0
C7—C8—C9	123.1 (3)	N1—C24—H24B	109.0
C7—C8—C13	118.3 (3)	C25—C24—H24B	109.0
C9—C8—C13	118.6 (3)	H24A—C24—H24B	107.8
C10—C9—C8	121.6 (3)	C24—C25—C26	113.0 (3)
C10—C9—H6	119.2	C24—C25—H25A	109.0
C8—C9—H6	119.2	C26—C25—H25A	109.0
C9—C10—C11	120.1 (3)	C24—C25—H25B	109.0
C9—C10—H10	119.9	C26—C25—H25B	109.0
C11—C10—H10	119.9	H25A—C25—H25B	107.8
C12—C11—C10	120.7 (3)	C27—C26—C25	109.6 (2)
C12—C11—H11	119.7	C27—C26—H26A	109.8
C10—C11—H11	119.7	C25—C26—H26A	109.8
C11—C12—C13	121.7 (3)	C27—C26—H26B	109.8
C11—C12—H12	119.2	C25—C26—H26B	109.8
C13—C12—H12	119.2	H26A—C26—H26B	108.2
C14—C13—C12	123.0 (2)	C28—C27—C26	111.7 (3)
C14—C13—C8	119.8 (2)	C28—C27—H27A	109.3
C12—C13—C8	117.3 (3)	C26—C27—H27A	109.3
C1—C14—C13	121.1 (2)	C28—C27—H27B	109.3
C1—C14—C15	119.6 (2)	C26—C27—H27B	109.3
C13—C14—C15	119.2 (2)	H27A—C27—H27B	107.9
O1—C15—C16	121.0 (2)	N1—C28—C27	112.6 (2)
O1—C15—C14	118.7 (2)	N1—C28—H28A	109.1
C16—C15—C14	120.2 (2)	C27—C28—H28A	109.1
C17—C16—C15	124.3 (2)	N1—C28—H28B	109.1
C17—C16—H16	117.9	C27—C28—H28B	109.1
C15—C16—H16	117.9	H28A—C28—H28B	107.8
			
C14—C1—C2—C3	−179.0 (2)	C8—C13—C14—C15	−175.9 (2)
C6—C1—C2—C3	0.3 (4)	C1—C14—C15—O1	−77.6 (3)
C1—C2—C3—C4	−1.0 (5)	C13—C14—C15—O1	99.6 (3)
C2—C3—C4—C5	0.5 (5)	C1—C14—C15—C16	101.5 (3)
C3—C4—C5—C6	0.6 (5)	C13—C14—C15—C16	−81.3 (3)
C4—C5—C6—C7	178.3 (3)	O1—C15—C16—C17	169.2 (3)
C4—C5—C6—C1	−1.3 (4)	C14—C15—C16—C17	−9.8 (4)
C14—C1—C6—C7	0.6 (4)	C15—C16—C17—C18	−175.6 (2)
C2—C1—C6—C7	−178.8 (2)	C16—C17—C18—C19	171.5 (3)
C14—C1—C6—C5	−179.8 (2)	C16—C17—C18—C23	−7.2 (4)
C2—C1—C6—C5	0.8 (3)	C23—C18—C19—C20	1.1 (4)
C5—C6—C7—C8	−178.6 (2)	C17—C18—C19—C20	−177.7 (3)
C1—C6—C7—C8	1.0 (4)	C18—C19—C20—C21	0.5 (5)
C6—C7—C8—C9	178.3 (2)	C24—N1—C21—C20	−5.5 (4)
C6—C7—C8—C13	−1.4 (4)	C28—N1—C21—C20	−146.8 (3)
C7—C8—C9—C10	178.1 (3)	C24—N1—C21—C22	176.8 (2)
C13—C8—C9—C10	−2.1 (4)	C28—N1—C21—C22	35.5 (3)
C8—C9—C10—C11	0.9 (4)	C19—C20—C21—N1	−179.3 (2)
C9—C10—C11—C12	0.7 (5)	C19—C20—C21—C22	−1.5 (4)
C10—C11—C12—C13	−0.9 (4)	N1—C21—C22—C23	178.8 (2)
C11—C12—C13—C14	−179.0 (2)	C20—C21—C22—C23	0.9 (4)
C11—C12—C13—C8	−0.4 (4)	C21—C22—C23—C18	0.7 (4)
C7—C8—C13—C14	0.3 (3)	C19—C18—C23—C22	−1.7 (4)
C9—C8—C13—C14	−179.4 (2)	C17—C18—C23—C22	177.1 (2)
C7—C8—C13—C12	−178.4 (2)	C21—N1—C24—C25	165.5 (2)
C9—C8—C13—C12	1.8 (3)	C28—N1—C24—C25	−51.5 (3)
C2—C1—C14—C13	177.7 (2)	N1—C24—C25—C26	51.8 (4)
C6—C1—C14—C13	−1.7 (3)	C24—C25—C26—C27	−52.4 (4)
C2—C1—C14—C15	−5.2 (4)	C25—C26—C27—C28	53.5 (4)
C6—C1—C14—C15	175.5 (2)	C21—N1—C28—C27	−163.3 (2)
C12—C13—C14—C1	179.9 (2)	C24—N1—C28—C27	53.3 (3)
C8—C13—C14—C1	1.2 (4)	C26—C27—C28—N1	−54.9 (4)
C12—C13—C14—C15	2.7 (4)		

### (E)-1-(Anthracen-9-yl)-3-[4-(piperidin-1-yl)phenyl]prop-2-en-1-one (I) Hydrogen-bond geometry (Å, º)

Cg1 is the centroid of the C18–C23 ring.

**Table:**

*D*—H···*A*	*D*—H	H···*A*	*D*···*A*	*D*—H···*A*
C28—H28*B*···O1^i^	0.97	2.36	3.262 (4)	154
C28—H28*A*···*Cg*1^ii^	0.97	2.95	3.861 (4)	157

Symmetry codes: (i) −*x*, −*y*+1, −*z*+2; (ii) −*x*+1, −*y*+1, −*z*+2.

### (E)-1-(Anthracen-9-yl)-3-[4-(diphenylamino)phenyl]prop-2-en-1-one (II) Crystal data

**Table:**

C_35_H_25_NO	*F*(000) = 2000
*M**_r_* = 475.56	*D*_x_ = 1.232 Mg m^−^^3^
Monoclinic, *C*2/*c*	Mo *K*α radiation, λ = 0.71073 Å
*a* = 31.2875 (16) Å	Cell parameters from 9835 reflections
*b* = 9.0470 (4) Å	θ = 2.3–21.9°
*c* = 18.3643 (8) Å	µ = 0.07 mm^−^^1^
β = 99.388 (3)°	*T* = 296 K
*V* = 5128.5 (4) Å^3^	Block, yellow
*Z* = 8	0.96 × 0.23 × 0.17 mm

### (E)-1-(Anthracen-9-yl)-3-[4-(diphenylamino)phenyl]prop-2-en-1-one (II) Data collection

**Table:**

Bruker SMART APEXII DUO CCD area-detector diffractometer	4183 reflections with *I* > 2σ(*I*)
Radiation source: fine-focus sealed tube	*R*_int_ = 0.076
φ and ω scans	θ_max_ = 30.4°, θ_min_ = 1.3°
Absorption correction: multi-scan (*SADABS*; Bruker, 2009)	*h* = −44→44
*T*_min_ = 0.645, *T*_max_ = 0.957	*k* = −12→12
98729 measured reflections	*l* = −26→26
7726 independent reflections	

### (E)-1-(Anthracen-9-yl)-3-[4-(diphenylamino)phenyl]prop-2-en-1-one (II) Refinement

**Table:**

Refinement on *F*^2^	0 restraints
Least-squares matrix: full	Hydrogen site location: inferred from neighbouring sites
*R*[*F*^2^ > 2σ(*F*^2^)] = 0.057	H-atom parameters constrained
*wR*(*F*^2^) = 0.144	*w* = 1/[σ^2^(*F*_o_^2^) + (0.0415*P*)^2^ + 2.2393*P*] where *P* = (*F*_o_^2^ + 2*F*_c_^2^)/3
*S* = 1.01	(Δ/σ)_max_ = 0.001
7726 reflections	Δρ_max_ = 0.13 e Å^−^^3^
334 parameters	Δρ_min_ = −0.14 e Å^−^^3^

### (E)-1-(Anthracen-9-yl)-3-[4-(diphenylamino)phenyl]prop-2-en-1-one (II) Special details

**Table:**

Experimental. The following wavelength and cell were deduced by SADABS from the direction cosines etc. They are given here for emergency use only: CELL 0.71163 9.142 16.459 18.559 99.001 89.988 106.089
Geometry. All esds (except the esd in the dihedral angle between two l.s. planes) are estimated using the full covariance matrix. The cell esds are taken into account individually in the estimation of esds in distances, angles and torsion angles; correlations between esds in cell parameters are only used when they are defined by crystal symmetry. An approximate (isotropic) treatment of cell esds is used for estimating esds involving l.s. planes.

### (E)-1-(Anthracen-9-yl)-3-[4-(diphenylamino)phenyl]prop-2-en-1-one (II) Fractional atomic coordinates and isotropic or equivalent isotropic displacement parameters (Å^2^)

**Table:**

	*x*	*y*	*z*	*U*_iso_*/*U*_eq_	
N1	0.54087 (4)	0.92358 (14)	0.60195 (7)	0.0596 (3)	
O1	0.76241 (4)	0.60494 (16)	0.40875 (8)	0.0900 (4)	
C1	0.69686 (5)	0.56889 (18)	0.25826 (8)	0.0548 (4)	
C2	0.70542 (5)	0.7199 (2)	0.24498 (10)	0.0667 (4)	
H2A	0.7163	0.7808	0.2845	0.080*	
C3	0.69796 (6)	0.7766 (3)	0.17592 (12)	0.0841 (6)	
H3A	0.7036	0.8759	0.1684	0.101*	
C4	0.68185 (8)	0.6870 (3)	0.11597 (12)	0.0993 (7)	
H4A	0.6766	0.7273	0.0688	0.119*	
C5	0.67379 (8)	0.5439 (3)	0.12545 (11)	0.0930 (7)	
H5A	0.6634	0.4860	0.0845	0.112*	
C6	0.68065 (6)	0.4783 (2)	0.19662 (9)	0.0677 (5)	
C7	0.67269 (6)	0.3305 (2)	0.20807 (11)	0.0783 (5)	
H7A	0.6621	0.2718	0.1675	0.094*	
C8	0.67976 (6)	0.26679 (19)	0.27691 (10)	0.0662 (5)	
C9	0.67272 (7)	0.1140 (2)	0.28858 (15)	0.0909 (7)	
H9A	0.6619	0.0544	0.2485	0.109*	
C10	0.68128 (8)	0.0541 (2)	0.35592 (16)	0.0987 (7)	
H10A	0.6765	−0.0462	0.3622	0.118*	
C11	0.69737 (7)	0.1414 (2)	0.41678 (13)	0.0858 (6)	
H11A	0.7036	0.0983	0.4633	0.103*	
C12	0.70399 (6)	0.2875 (2)	0.40906 (10)	0.0681 (5)	
H12A	0.7141	0.3442	0.4505	0.082*	
C13	0.69584 (5)	0.35568 (17)	0.33898 (9)	0.0545 (4)	
C14	0.70393 (4)	0.50520 (17)	0.32830 (8)	0.0504 (3)	
C15	0.72316 (5)	0.59679 (18)	0.39314 (8)	0.0554 (4)	
C16	0.69564 (5)	0.67286 (17)	0.43679 (8)	0.0553 (4)	
H16A	0.7088	0.7352	0.4741	0.066*	
C17	0.65301 (5)	0.66021 (16)	0.42750 (8)	0.0506 (3)	
H17A	0.6402	0.5991	0.3894	0.061*	
C18	0.62422 (5)	0.73214 (16)	0.47080 (7)	0.0484 (3)	
C19	0.58198 (5)	0.68422 (18)	0.46718 (8)	0.0568 (4)	
H19A	0.5719	0.6082	0.4348	0.068*	
C20	0.55459 (5)	0.74561 (18)	0.50998 (9)	0.0585 (4)	
H20A	0.5264	0.7100	0.5065	0.070*	
C21	0.56838 (5)	0.86064 (16)	0.55852 (8)	0.0498 (3)	
C22	0.61055 (5)	0.91102 (16)	0.56133 (8)	0.0511 (3)	
H22A	0.6205	0.9887	0.5927	0.061*	
C23	0.63763 (5)	0.84824 (16)	0.51872 (8)	0.0508 (3)	
H23A	0.6657	0.8841	0.5219	0.061*	
C24	0.50401 (5)	0.84596 (19)	0.61751 (8)	0.0561 (4)	
C25	0.50740 (6)	0.7044 (2)	0.64455 (10)	0.0742 (5)	
H25A	0.5343	0.6582	0.6533	0.089*	
C26	0.47145 (8)	0.6306 (3)	0.65871 (12)	0.0934 (7)	
H26A	0.4740	0.5341	0.6764	0.112*	
C27	0.43226 (8)	0.6972 (3)	0.64707 (13)	0.1015 (8)	
H27A	0.4079	0.6470	0.6568	0.122*	
C28	0.42867 (7)	0.8379 (3)	0.62104 (12)	0.0937 (7)	
H28A	0.4017	0.8837	0.6132	0.112*	
C29	0.46430 (6)	0.9135 (2)	0.60614 (9)	0.0714 (5)	
H29A	0.4615	1.0099	0.5885	0.086*	
C30	0.55076 (5)	1.06241 (18)	0.63734 (9)	0.0572 (4)	
C31	0.55374 (7)	1.0733 (2)	0.71230 (10)	0.0883 (6)	
H31A	0.5491	0.9905	0.7400	0.106*	
C32	0.56357 (9)	1.2064 (3)	0.74657 (13)	0.1146 (9)	
H32A	0.5654	1.2134	0.7975	0.137*	
C33	0.57072 (8)	1.3275 (3)	0.70722 (16)	0.1042 (8)	
H33A	0.5779	1.4168	0.7311	0.125*	
C34	0.56734 (7)	1.3186 (2)	0.63278 (13)	0.0865 (6)	
H34A	0.5718	1.4023	0.6056	0.104*	
C35	0.55728 (6)	1.18625 (19)	0.59741 (10)	0.0695 (5)	
H35A	0.5549	1.1806	0.5463	0.083*	

### (E)-1-(Anthracen-9-yl)-3-[4-(diphenylamino)phenyl]prop-2-en-1-one (II) Atomic displacement parameters (Å^2^)

**Table:**

	*U*^11^	*U*^22^	*U*^33^	*U*^12^	*U*^13^	*U*^23^
N1	0.0647 (8)	0.0574 (8)	0.0621 (8)	−0.0005 (6)	0.0262 (6)	−0.0097 (6)
O1	0.0500 (7)	0.1216 (11)	0.0971 (10)	−0.0044 (7)	0.0079 (6)	−0.0522 (9)
C1	0.0488 (8)	0.0628 (9)	0.0562 (9)	−0.0018 (7)	0.0182 (7)	−0.0101 (8)
C2	0.0596 (10)	0.0703 (11)	0.0742 (11)	−0.0030 (8)	0.0227 (8)	−0.0012 (9)
C3	0.0780 (13)	0.0912 (15)	0.0907 (15)	0.0032 (11)	0.0360 (11)	0.0192 (12)
C4	0.1056 (17)	0.131 (2)	0.0662 (13)	0.0071 (16)	0.0295 (12)	0.0171 (14)
C5	0.1064 (17)	0.1208 (19)	0.0534 (11)	−0.0031 (15)	0.0174 (10)	−0.0125 (12)
C6	0.0677 (11)	0.0839 (13)	0.0533 (10)	−0.0038 (9)	0.0155 (8)	−0.0155 (9)
C7	0.0864 (13)	0.0825 (13)	0.0673 (12)	−0.0145 (10)	0.0162 (10)	−0.0346 (10)
C8	0.0661 (10)	0.0614 (10)	0.0748 (12)	−0.0072 (8)	0.0226 (9)	−0.0239 (9)
C9	0.1045 (16)	0.0618 (12)	0.1124 (18)	−0.0143 (11)	0.0356 (14)	−0.0324 (12)
C10	0.1129 (18)	0.0577 (12)	0.136 (2)	−0.0023 (12)	0.0523 (16)	−0.0029 (14)
C11	0.0895 (14)	0.0722 (13)	0.1014 (16)	0.0072 (11)	0.0322 (12)	0.0109 (12)
C12	0.0664 (11)	0.0692 (11)	0.0717 (11)	0.0008 (9)	0.0200 (9)	−0.0027 (9)
C13	0.0498 (8)	0.0561 (9)	0.0607 (9)	−0.0017 (7)	0.0187 (7)	−0.0112 (8)
C14	0.0435 (7)	0.0578 (9)	0.0522 (8)	−0.0015 (6)	0.0145 (6)	−0.0144 (7)
C15	0.0483 (8)	0.0612 (9)	0.0571 (9)	−0.0016 (7)	0.0097 (7)	−0.0127 (7)
C16	0.0543 (9)	0.0608 (9)	0.0505 (8)	−0.0010 (7)	0.0077 (7)	−0.0166 (7)
C17	0.0549 (9)	0.0534 (8)	0.0434 (8)	−0.0002 (7)	0.0080 (6)	−0.0074 (6)
C18	0.0504 (8)	0.0520 (8)	0.0429 (7)	0.0036 (6)	0.0077 (6)	−0.0048 (6)
C19	0.0553 (9)	0.0608 (9)	0.0545 (9)	−0.0031 (7)	0.0090 (7)	−0.0154 (7)
C20	0.0509 (8)	0.0650 (10)	0.0611 (9)	−0.0042 (7)	0.0133 (7)	−0.0117 (8)
C21	0.0547 (8)	0.0525 (8)	0.0438 (8)	0.0059 (7)	0.0128 (6)	−0.0022 (6)
C22	0.0566 (9)	0.0511 (8)	0.0448 (8)	0.0026 (7)	0.0058 (6)	−0.0077 (6)
C23	0.0495 (8)	0.0543 (8)	0.0486 (8)	0.0001 (7)	0.0080 (6)	−0.0050 (7)
C24	0.0606 (9)	0.0653 (10)	0.0448 (8)	−0.0003 (8)	0.0161 (7)	−0.0058 (7)
C25	0.0765 (12)	0.0699 (12)	0.0784 (12)	−0.0023 (9)	0.0189 (9)	0.0062 (10)
C26	0.1081 (18)	0.0881 (15)	0.0889 (15)	−0.0294 (14)	0.0308 (13)	−0.0025 (12)
C27	0.0915 (17)	0.130 (2)	0.0908 (16)	−0.0422 (16)	0.0377 (13)	−0.0292 (15)
C28	0.0591 (12)	0.134 (2)	0.0910 (15)	−0.0019 (13)	0.0198 (10)	−0.0251 (15)
C29	0.0676 (11)	0.0879 (13)	0.0604 (10)	0.0107 (10)	0.0152 (8)	−0.0034 (9)
C30	0.0609 (9)	0.0586 (9)	0.0549 (9)	0.0057 (7)	0.0179 (7)	−0.0100 (8)
C31	0.1208 (17)	0.0893 (14)	0.0589 (11)	−0.0092 (13)	0.0272 (11)	−0.0114 (10)
C32	0.155 (2)	0.119 (2)	0.0749 (15)	−0.0213 (18)	0.0321 (15)	−0.0418 (15)
C33	0.1124 (18)	0.0869 (16)	0.118 (2)	−0.0103 (14)	0.0336 (15)	−0.0477 (15)
C34	0.0954 (15)	0.0605 (11)	0.1077 (17)	0.0056 (10)	0.0284 (12)	−0.0109 (11)
C35	0.0818 (12)	0.0621 (11)	0.0662 (11)	0.0100 (9)	0.0166 (9)	−0.0025 (9)

### (E)-1-(Anthracen-9-yl)-3-[4-(diphenylamino)phenyl]prop-2-en-1-one (II) Geometric parameters (Å, º)

**Table:**

N1—C21	1.3876 (18)	C17—H17A	0.9300
N1—C24	1.419 (2)	C18—C19	1.382 (2)
N1—C30	1.425 (2)	C18—C23	1.390 (2)
O1—C15	1.2170 (18)	C19—C20	1.371 (2)
C1—C14	1.394 (2)	C19—H19A	0.9300
C1—C2	1.421 (2)	C20—C21	1.392 (2)
C1—C6	1.422 (2)	C20—H20A	0.9300
C2—C3	1.353 (3)	C21—C22	1.389 (2)
C2—H2A	0.9300	C22—C23	1.3672 (19)
C3—C4	1.394 (3)	C22—H22A	0.9300
C3—H3A	0.9300	C23—H23A	0.9300
C4—C5	1.336 (3)	C24—C29	1.370 (2)
C4—H4A	0.9300	C24—C25	1.372 (2)
C5—C6	1.420 (3)	C25—C26	1.369 (3)
C5—H5A	0.9300	C25—H25A	0.9300
C6—C7	1.382 (3)	C26—C27	1.352 (3)
C7—C8	1.374 (3)	C26—H26A	0.9300
C7—H7A	0.9300	C27—C28	1.358 (3)
C8—C13	1.419 (2)	C27—H27A	0.9300
C8—C9	1.422 (3)	C28—C29	1.373 (3)
C9—C10	1.337 (3)	C28—H28A	0.9300
C9—H9A	0.9300	C29—H29A	0.9300
C10—C11	1.394 (3)	C30—C31	1.368 (2)
C10—H10A	0.9300	C30—C35	1.372 (2)
C11—C12	1.349 (3)	C31—C32	1.370 (3)
C11—H11A	0.9300	C31—H31A	0.9300
C12—C13	1.412 (2)	C32—C33	1.351 (3)
C12—H12A	0.9300	C32—H32A	0.9300
C13—C14	1.396 (2)	C33—C34	1.356 (3)
C14—C15	1.494 (2)	C33—H33A	0.9300
C15—C16	1.443 (2)	C34—C35	1.374 (3)
C16—C17	1.322 (2)	C34—H34A	0.9300
C16—H16A	0.9300	C35—H35A	0.9300
C17—C18	1.4494 (19)		
			
C21—N1—C24	120.91 (13)	C19—C18—C23	117.05 (13)
C21—N1—C30	121.00 (13)	C19—C18—C17	120.52 (13)
C24—N1—C30	117.88 (12)	C23—C18—C17	122.41 (13)
C14—C1—C2	123.36 (15)	C20—C19—C18	121.84 (14)
C14—C1—C6	118.58 (15)	C20—C19—H19A	119.1
C2—C1—C6	118.06 (16)	C18—C19—H19A	119.1
C3—C2—C1	121.13 (18)	C19—C20—C21	120.81 (14)
C3—C2—H2A	119.4	C19—C20—H20A	119.6
C1—C2—H2A	119.4	C21—C20—H20A	119.6
C2—C3—C4	120.3 (2)	N1—C21—C22	121.24 (13)
C2—C3—H3A	119.8	N1—C21—C20	121.20 (14)
C4—C3—H3A	119.8	C22—C21—C20	117.55 (13)
C5—C4—C3	120.8 (2)	C23—C22—C21	121.07 (14)
C5—C4—H4A	119.6	C23—C22—H22A	119.5
C3—C4—H4A	119.6	C21—C22—H22A	119.5
C4—C5—C6	121.5 (2)	C22—C23—C18	121.66 (14)
C4—C5—H5A	119.3	C22—C23—H23A	119.2
C6—C5—H5A	119.3	C18—C23—H23A	119.2
C7—C6—C5	122.72 (18)	C29—C24—C25	119.11 (17)
C7—C6—C1	119.09 (17)	C29—C24—N1	119.71 (16)
C5—C6—C1	118.19 (18)	C25—C24—N1	121.19 (15)
C8—C7—C6	122.66 (16)	C26—C25—C24	120.5 (2)
C8—C7—H7A	118.7	C26—C25—H25A	119.8
C6—C7—H7A	118.7	C24—C25—H25A	119.8
C7—C8—C13	118.97 (16)	C27—C26—C25	120.3 (2)
C7—C8—C9	122.75 (18)	C27—C26—H26A	119.9
C13—C8—C9	118.26 (19)	C25—C26—H26A	119.9
C10—C9—C8	121.4 (2)	C26—C27—C28	119.7 (2)
C10—C9—H9A	119.3	C26—C27—H27A	120.2
C8—C9—H9A	119.3	C28—C27—H27A	120.2
C9—C10—C11	120.3 (2)	C27—C28—C29	120.9 (2)
C9—C10—H10A	119.9	C27—C28—H28A	119.5
C11—C10—H10A	119.9	C29—C28—H28A	119.5
C12—C11—C10	120.8 (2)	C24—C29—C28	119.6 (2)
C12—C11—H11A	119.6	C24—C29—H29A	120.2
C10—C11—H11A	119.6	C28—C29—H29A	120.2
C11—C12—C13	121.06 (18)	C31—C30—C35	119.13 (17)
C11—C12—H12A	119.5	C31—C30—N1	119.80 (16)
C13—C12—H12A	119.5	C35—C30—N1	121.07 (14)
C14—C13—C12	122.79 (15)	C30—C31—C32	120.0 (2)
C14—C13—C8	119.02 (15)	C30—C31—H31A	120.0
C12—C13—C8	118.17 (15)	C32—C31—H31A	120.0
C1—C14—C13	121.68 (14)	C33—C32—C31	120.7 (2)
C1—C14—C15	119.26 (14)	C33—C32—H32A	119.6
C13—C14—C15	118.95 (14)	C31—C32—H32A	119.6
O1—C15—C16	120.72 (14)	C32—C33—C34	119.9 (2)
O1—C15—C14	118.75 (13)	C32—C33—H33A	120.1
C16—C15—C14	120.53 (13)	C34—C33—H33A	120.1
C17—C16—C15	124.60 (14)	C33—C34—C35	120.2 (2)
C17—C16—H16A	117.7	C33—C34—H34A	119.9
C15—C16—H16A	117.7	C35—C34—H34A	119.9
C16—C17—C18	126.49 (14)	C30—C35—C34	120.09 (18)
C16—C17—H17A	116.8	C30—C35—H35A	120.0
C18—C17—H17A	116.8	C34—C35—H35A	120.0
			
C14—C1—C2—C3	−179.93 (15)	C16—C17—C18—C19	−164.77 (16)
C6—C1—C2—C3	0.7 (2)	C16—C17—C18—C23	13.6 (2)
C1—C2—C3—C4	−0.4 (3)	C23—C18—C19—C20	−1.4 (2)
C2—C3—C4—C5	−0.5 (3)	C17—C18—C19—C20	176.98 (15)
C3—C4—C5—C6	0.9 (4)	C18—C19—C20—C21	0.7 (3)
C4—C5—C6—C7	−179.8 (2)	C24—N1—C21—C22	−159.50 (14)
C4—C5—C6—C1	−0.6 (3)	C30—N1—C21—C22	15.1 (2)
C14—C1—C6—C7	−0.3 (2)	C24—N1—C21—C20	21.1 (2)
C2—C1—C6—C7	179.07 (16)	C30—N1—C21—C20	−164.28 (15)
C14—C1—C6—C5	−179.64 (16)	C19—C20—C21—N1	179.90 (15)
C2—C1—C6—C5	−0.2 (2)	C19—C20—C21—C22	0.5 (2)
C5—C6—C7—C8	179.33 (18)	N1—C21—C22—C23	179.68 (14)
C1—C6—C7—C8	0.1 (3)	C20—C21—C22—C23	−0.9 (2)
C6—C7—C8—C13	0.1 (3)	C21—C22—C23—C18	0.2 (2)
C6—C7—C8—C9	−178.22 (18)	C19—C18—C23—C22	1.0 (2)
C7—C8—C9—C10	177.7 (2)	C17—C18—C23—C22	−177.38 (14)
C13—C8—C9—C10	−0.6 (3)	C21—N1—C24—C29	−129.43 (16)
C8—C9—C10—C11	0.2 (3)	C30—N1—C24—C29	55.8 (2)
C9—C10—C11—C12	0.9 (3)	C21—N1—C24—C25	51.2 (2)
C10—C11—C12—C13	−1.6 (3)	C30—N1—C24—C25	−123.52 (17)
C11—C12—C13—C14	−177.34 (16)	C29—C24—C25—C26	1.2 (3)
C11—C12—C13—C8	1.1 (2)	N1—C24—C25—C26	−179.45 (16)
C7—C8—C13—C14	0.1 (2)	C24—C25—C26—C27	−0.9 (3)
C9—C8—C13—C14	178.47 (16)	C25—C26—C27—C28	0.2 (3)
C7—C8—C13—C12	−178.37 (16)	C26—C27—C28—C29	0.2 (3)
C9—C8—C13—C12	0.0 (2)	C25—C24—C29—C28	−0.8 (3)
C2—C1—C14—C13	−178.85 (14)	N1—C24—C29—C28	179.80 (16)
C6—C1—C14—C13	0.5 (2)	C27—C28—C29—C24	0.2 (3)
C2—C1—C14—C15	−2.6 (2)	C21—N1—C30—C31	−123.00 (18)
C6—C1—C14—C15	176.76 (14)	C24—N1—C30—C31	51.7 (2)
C12—C13—C14—C1	178.00 (14)	C21—N1—C30—C35	57.2 (2)
C8—C13—C14—C1	−0.4 (2)	C24—N1—C30—C35	−128.10 (17)
C12—C13—C14—C15	1.7 (2)	C35—C30—C31—C32	−0.7 (3)
C8—C13—C14—C15	−176.67 (13)	N1—C30—C31—C32	179.5 (2)
C1—C14—C15—O1	−87.0 (2)	C30—C31—C32—C33	−0.5 (4)
C13—C14—C15—O1	89.39 (19)	C31—C32—C33—C34	1.3 (4)
C1—C14—C15—C16	93.66 (18)	C32—C33—C34—C35	−0.9 (4)
C13—C14—C15—C16	−89.99 (18)	C31—C30—C35—C34	1.0 (3)
O1—C15—C16—C17	−174.13 (17)	N1—C30—C35—C34	−179.15 (16)
C14—C15—C16—C17	5.2 (3)	C33—C34—C35—C30	−0.2 (3)
C15—C16—C17—C18	178.77 (15)		

### (E)-1-(Anthracen-9-yl)-3-[4-(diphenylamino)phenyl]prop-2-en-1-one (II) Hydrogen-bond geometry (Å, º)

Cg1 is the centroid of the C18–C23 ring.

**Table:**

*D*—H···*A*	*D*—H	H···*A*	*D*···*A*	*D*—H···*A*
C23—H23*A*···O1^i^	0.93	2.40	3.221 (2)	147
C29—H29*A*···*Cg*1^ii^	0.93	2.96	3.739 (19)	142

Symmetry codes: (i) −*x*+3/2, −*y*+3/2, −*z*+1; (ii) *x*+3/2, *y*+5/2, *z*+1.
