# Supplementary material for: Mol­ecular structure, DFT studies and Hirshfeld analysis of anthracenyl chalcone derivatives
Source: Acta Crystallogr E Crystallogr Commun. 2018 May 4;74(Pt 6):780–5. doi: 10.1107/S2056989018006527 (PMC6002829; doi:10.1107/S2056989018006527)
Supplement: Supplementary file 4 [file e-74-00780-sup4.pdf]

Molecular structure, DFT studies and Hirshfeld analysis of the anthracenyl chalcones derivative

Supplementary Materials

Table S1 Comparison between calculated (DFT) and X-ray of selected geometrical data for title compound

	Compound I		Compound II	
**Bonds**	**X-ray (Experimental) (Å^0)**	**DFT (Theoretical) (Å^0)**	**X-ray (Experimental) (Å^0)**	**DFT (Theoretical) (Å^0)**
C15-O1	1.22 (3)	1.22	1.22 (18)	1.22
C1-C14	1.39 (3)	1.41	1.39 (2)	1.41
C1-C2	1.42 (3)	1.43	1.42 (2)	1.43
C2-C3	1.35 (4)	1.37	1.35 (3)	1.37
C3-C4	1.40 (4)	1.42	1.39 (3)	1.42
C4-C5	1.34 (4)	1.37	1.33 (3)	1.37
C5-C6	1.42 (4)	1.43	1.42 (3)	1.43
C6-C7	1.38 (4)	1.39	1.38 (3)	1.39
C7-C8	1.38 (4)	1.39	1.37 (3)	1.39
C8-C9	1.41 (4)	1.43	1.42 (3)	1.43
C9-C10	1.33 (4)	1.37	1.34 (3)	1.37
C10-C11	1.41 (4)	1.42	1.39 (3)	1.42
C11-C12	1.35 (4)	1.37	1.35 (3)	1.37
C12-C13	1.42 (4)	1.43	1.41 (2)	1.43
C13-C14	1.39 (3)	1.41	1.40 (2)	1.41
C14-C15	1.50 (3)	1.52	1.49 (2)	1.52
C15-C16	1.44 (3)	1.47	1.44 (2)	1.47
C16-C17	1.32 (3)	1.35	1.32 (2)	1.35
C17-C18	1.44 (3)	1.45	1.45 (19)	1.45
C14—C15—C16	120.2 (2)	119.43	120.53 (13)	119.33
O1—C15—C14	118.7 (2)	119.61	118.75 (13)	119.80
O1—C15—C16	121.0 (2)	120.97	120.72 (14)	120.87
C15—C16—C17	124.3 (2)	124.21	124.60 (14)	124.14
C16—C17—C18	128.4 (2)	127.76	126.49 (14)	127.74
